# Expected and Experienced Social Impact of Host Residents During Rugby World Cup 2019: A Panel Data Approach

**DOI:** 10.3389/fspor.2021.628153

**Published:** 2021-02-11

**Authors:** Daichi Oshimi, Shiro Yamaguchi, Takayuki Fukuhara, Marijke Taks

**Affiliations:** ^1^School of Physical Education, Tokai University, Tokyo, Japan; ^2^Faculty of Human and Social Sciences, University of Marketing and Distribution Sciences, Kobe, Japan; ^3^Art and Sports Business Course, Hokkaido University of Education, Hokkaido, Japan; ^4^School of Human Kinetics, University of Ottawa, Ottawa, ON, Canada

**Keywords:** expected social impact, experienced social impact, host residents, viewing behavior, panel data approach, sport events

## Abstract

Most social impact research considers the pre- and post-event social impacts of sporting events to investigate the effects of these events on residents' or consumers' intention or attitude. This study focused on the qualitative differences between pre-event expected social impacts (T1) and post-event experienced social impacts (T2). Then, it investigated viewing behaviors due to the expected social impacts, and intentions to support events from experienced social impacts. The Rugby World Cup 2019 in Japan provided the context for the study. Panel data were collected from the same Tokyo residents in T1 (3 months before the event) and T2 (4 months after the event). The Internet-based survey consisted of six social impact constructs, framed as expectations in T1 and experiences in T2. Both dependent variables, viewing behavior and supporting events, were measured in T2, after the event occurred. Two expected impacts had a significant positive association with viewing behavior, while three experienced social impacts had a significant positive association with event support intention. The main contribution of this article is extending the understanding of the role of social impact as a predictor variable for residents' behavior and intention to support events by using panel data, which enabled the authors to obtain more robust results. The current study extends the knowledge on consumer expectancy role and social exchange theory in the context of the social impacts of sporting events.

## Introduction

Over the past decade, social impact studies have informed policy makers and/or event organizers to consider the justification of organizing sporting events. In particular, major sporting events should be hosted with the consent of the local residents by validating their benefits and minimizing disorder or congestion in the hosting area, especially if public funds are required to support the event (Ohmann et al., 2006). Most studies in this area help shed light on the social value of sporting events (e.g., Waitt, 2003; Ritchie et al., 2009; Balduck et al., 2011), as well as the economic impact of sports (e.g., Crompton et al., 2001; Preuss, 2005; Agha and Taks, 2015). Typical social impact research compares pre- and post-social impact evaluations of the event (e.g., Kim et al., 2006; Heere et al., 2013; Gibson et al., 2014). This approach is derived from the definition of social impact, such as the changes in the collective and individual value or behavior patterns due to tourism or travel (Hall, 1992). As these “changes” are a key concept of social impact, many scholars have applied the pre- and post-event (or pre-, during, and post-event) comparison approach to identify the changes incurred by an event. The other major approach of social impact research is investigating the relationship between the impact perception and people's support intention toward the event (e.g., Gursoy and Kendall, 2006; Lee and Krohn, 2013; Pappas, 2014).

In the current study, the authors focused on the qualitative differences between pre-event social impact expectations (in T1) and post-event social impact experiences (in T2). In other words, before an event is hosted, residents can only anticipate their possible experiences as the event has not yet occurred. Hence, they can only express their expectations or guess on the possible positive and negative impacts (i.e., expected social impacts) of the event. Therefore, the associated benefits or costs are weighted based on what they think will happen, which will consequently inform their positive and/or negative attitude toward the event. However, post-event, residents can evaluate their social impact experiences as the event has now occurred. Thus, the quality of the pre-event expected and the post-event experienced social impacts might be different, similar to the expectancy disconfirmation paradigm. This paradigm considers that the difference between the expectation and perceived performance of service qualities affects consumer evaluation (Oliver, 2010). In the current study, the authors defined pre-event social impact as an “expected social impact” and the post-event social impact as an “experienced social impact” by assuming that pre- and post-event social impacts have different effects on residents' responses to the event. Specifically, this study constructs a behavioral indicator (i.e., viewing behavior; measured in T2 when the behavior has possibly occurred) as a dependent variable of the expected social impact and the intention for event support (also measured in T2) as a dependent variable of each experienced social impact using a panel-data approach.

Through this analysis, the existing social impact literature is broadened by addressing the following previous limitations. First, despite some efforts to investigate the relationship between the expected social impact and residents' intention (e.g., Balduck et al., 2011; Oshimi et al., 2016), only few studies have clarified the relationship between the expected social impact and residents' behavior (e.g., viewing behavior), which is an actual behavioral outcome. Considering the limitation of the explanatory power of consumer intention to their actual behavior (Morwitz, 1997; Yoshida et al., 2013), applying behavioral variables in the analysis could strengthen the validity of the results. Second, although the literature has clarified the influence of perceived social impact on event support intention, many researchers have summarized the various factors and/or items into one general factor (e.g., perceived positive impacts) to investigate its influence on support intention (e.g., Gursoy and Kendall, 2006; Pappas, 2014; Al-Emadi et al., 2017). This prevents us from determining the more effective factors for their attitudes and/or intentions. Third, many previous studies on social impacts used language to capture social impacts in terms of a “generic other” based on their beliefs and opinions (i.e., using “they,” “their,” and “people”), rather than their own experiences. In this study, all items are based on experiences. These “self-referenced” items (i.e., using “me,” “my,” and “I”) offer a more valid perspective of social impacts (Taks et al., 2020). Fourth, some researchers have used repeated cross-sectional designs in two different time period (pre- and post-event) procedures (e.g., Kim and Petrick, 2005; Gibson et al., 2014). However, the number of social impact studies that used the same sample panel data is limited. By collecting data from the same subjects at both time intervals, a panel data approach can achieve more robust results than the cross-sectional design (Menard, 1991); this allows the verification of residents' actual behavior (viewing behavior, measured in T2) and comprehending residents' experiences related to the event.

Therefore, this study aims to evaluate the effect of pre- and post-event social impacts on residents' behavior and event support intention, respectively, through panel data by dividing social impact into expected and experienced social impacts. This is expected to contribute to the further understanding of the role of social impact as a determinant variable of residents' behavior and decision-making processes by their expectation and experience toward the event.

## Literature Review

### Social Impact of Sporting Events

The number of social impact studies has increased since the 1980s, especially those focusing on mega and/or major sporting events by applying pre- and post-event comparison to verify the changes in the impact perception of the event (e.g., Kim and Petrick, 2005; Balduck et al., 2011; Gibson et al., 2014). Other types of social impact research investigate the influence of social impact on residents' intentions (e.g., Kaplanidou et al., 2013; Prayag et al., 2013; Inoue and Havard, 2014). Several types of intentions have been utilized in research streams, such as positive word of mouth (Inoue and Havard, 2014), event support (Kaplanidou et al., 2013; Prayag et al., 2013; Parra-Camacho et al., 2020b), support for sports policy (Parra-Camacho et al., 2020d), and future intention to host the event (Liu, 2016; Oshimi et al., 2016; Parra-Camacho et al., 2020a). However, little evidence exists on the relationships between social impact and actual residents' behavior (i.e., viewing behavior). As viewing behavior is a major type of sports consumption (e.g., Gantz and Wenner, 1995), clarifying this relationship is highly relevant for marketeers and enhances our understanding of the role of social impacts on sporting events.

The number of social impact studies using same sample panel data is limited. Although some researchers have used repeated cross-sectional designs, such as the pre- and post-event procedure (e.g., Kim and Petrick, 2005; Kaplanidou et al., 2013; Gibson et al., 2014), the statistical robustness of these approaches is insufficient (Ritchie et al., 2009). Further, valid approaches that apply a pure longitudinal (panel data) approach that combines cross-sectional (at a single point in time with more than one set of sample data) and longitudinal data (at more than a single point in time with one set of sample data) are rare (Oshimi et al., 2016), owing to the associated challenges (e.g., time and resources; Ritchie et al., 2009). Therefore, the current study utilizes a panel data design to improve the robustness of research on social impact.

### Expected Social Impact

Based on expectancy theory (Vroom, 1964), action or behavior is motivated by knowledge and beliefs about outcomes and was developed to explain work motivation and behavior in the company. This cognitive theory explains the decision-making process, and the concept of expectancy describes a person's degree of belief as a predictor of consumer behavior (Feather, 1990). In other words, expectancy could anticipate a certain behavior based on its expected results (Hsu et al., 2010). Previous research applied this concept to explain consumer intention or attitude using expected outcomes, especially in the tourism literature (e.g., Hsu et al., 2010; Lee et al., 2011; Wong et al., 2013; Fan and Hsu, 2014). For example, Hsu et al. (2010) verified the positive relationship between customer expectations and positive attitudes toward a destination, and Lee et al. (2011) found a positive influence from expected outcomes of staying in hotels on behavioral intentions (e.g., visit and word-of-mouth intentions). The current research considers the expected social impact in pre-event as expectations (i.e., belief) for outcomes related to the event. Additionally, expectations are mainly constructed based on cognitive attributes, but they also include attitudinal factors, such as emotional dominant expectations (Gnoth, 1997). For example, previous research has shown that anticipated positive emotions are the dominant elements in the human decision-making process (Conner and Armitage, 1998; Perugini and Bagozzi, 2001; Yu, 2020).

In the social impact research context, the social impact of sporting events includes cognitive (e.g., sports participation development; Weed et al., 2015) and emotional value (e.g., psychic income; Oja et al., 2018). In particular, a growing body of literature emphasized the emotional value of sports in predicting sporting event attendance (Hall et al., 2010; Biscaia et al., 2012; Kim et al., 2017). Therefore, expectancy theory can be used in social impact research on sporting events to predict consumer behaviors. The relationship between expected benefits and behavior is also analyzed in the customer satisfaction literature (e.g., Oliver, 1977, 1980); this concludes that expectations stimulate the probability of future consequences, such as decision-making processes and purchase intentions (Oliver, 2010). Although social impact literature investigated the relationship between expected social impact and residents' intention (e.g., Balduck et al., 2011; Oshimi et al., 2016), few studies have clarified the influence on actual consumer behaviors. Therefore, the first research question this study addresses was developed to guide this investigation:

RQ1: Which expected social impacts influenced (positively/negatively) residents' viewing behavior for Rugby World Cup 2019?

### Experienced Social Impact

Previous research verified the relationship between perceived social impact and residents' intention to support a mega and/or major event (e.g., Gursoy and Kendall, 2006; Lee and Krohn, 2013; Pappas, 2014). In many cases, this causal relationship has been supported by the social exchange theory, which is “a general sociological theory concerned with understanding the exchange of resources between individuals and groups in an interaction situation” (Ap, 1992, p. 668). This theory, which is most commonly applied in social impact research (e.g., Waitt, 2003; Kim and Walker, 2012; Ma et al., 2013), assumes that individuals evaluate circumstances and experiences based on cost–benefit analyses. In other words, when the benefit of an event outweighs its cost, people form a positive attitude toward the event; conversely, if the cost outweighs the benefit, they show a negative attitude toward the event. The current research also utilized this theory to predict residents' intentions toward event support.

Although the literature has clarified the influence of perceived social impact on behavioral intention (e.g., event support intention) in the context of mega and major sporting events, many researchers have summarized the various factors and/or items into one general factor (e.g., perceived positive/negative impacts/benefits) to investigate its influence on support intention (e.g., Gursoy and Kendall, 2006; Lee and Krohn, 2013; Pappas, 2014; Gursoy et al., 2016; Al-Emadi et al., 2017; Ouyang et al., 2017). Other studies set a mediator variable (i.e., overall attitude or overall satisfaction) between each social impact and event support/future intention (e.g., Kaplanidou et al., 2013; Prayag et al., 2013; Parra-Camacho et al., 2020a; Zhang et al., 2020). While this approach is useful for simplifying the causal relationship between perceived social impact and consumer attitudes/intention, it prevents us from clarifying which factors are more effective for their attitudes/intention. Although a few studies have investigated the direct influence of each factor, such as cultural interest and consolidation (Balduck et al., 2011), image and status (Liu, 2016), and image and awareness (Lee and Krohn, 2013) on event support intention, the empirical evidence remains limited. Therefore, we need to understand what type of social impact is beneficial to residents' event support intention. Accordingly, the following research question was developed:

RQ2: Which experienced social impact influenced (positively/negatively) residents' event support intention for Rugby World Cup 2019?

**Figure 1:**
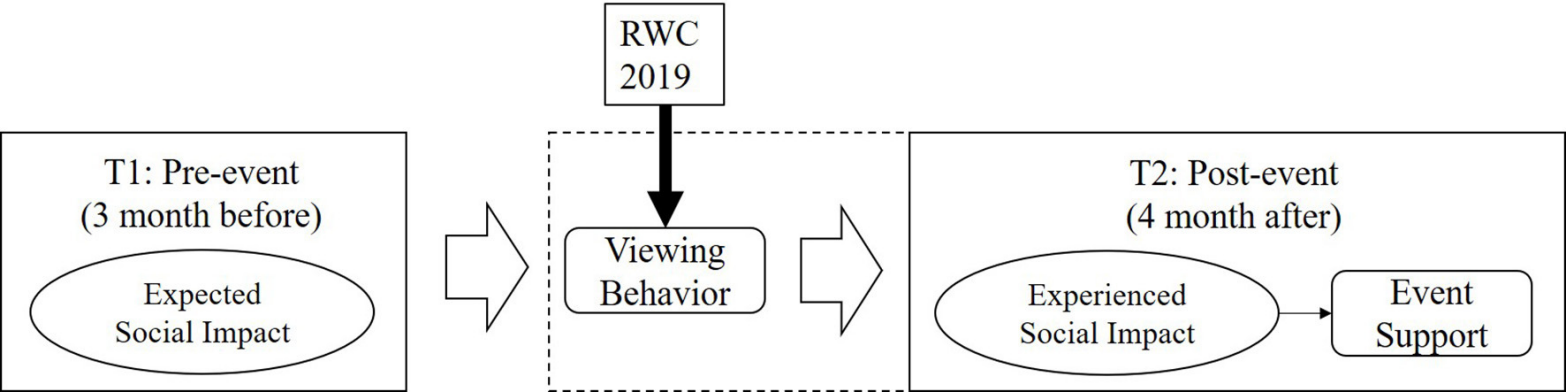
Conceptual model. Dotted line: variable collected in T2.

Figure 1 shows the conceptual model. The left-hand side represents residents' expected social impact 3 months before the event (T1) and the relationship between residents' viewing behavior, as well as the relationship between experienced social impacts and event support intention 4 months after the event (measured in T2). This approach can grasp the entire picture of social impacts on residents' behavior and/or intention by applying a panel data approach (i.e., the same sample in pre- and post-events). Furthermore, all items in the current research are worded in the “self-referenced” items (i.e., using “me,” “my,” and “I”), which offers a more valid perspective of social impacts (Taks et al., 2020). Most research has utilized “other-referenced” items (Balduck et al., 2011; Kim et al., 2015; Oshimi et al., 2016), such as “The event will make “people” feel strongly connected to one another.” Conversely, self-referenced items, such as “The event will make “me” feel strongly connected to one another,” provide a more accurate personal opinion toward the event.

## Methods

### Research Context (Rugby World Cup 2019)

The Rugby World Cup 2019 was held in Japan, being the first Rugby Union World Cup held in the Asian region. The event was a good opportunity for further development of rugby in Asia and provided significant marketing opportunities for the International Rugby Board (IRB) to reach the Asian market (Asia Rugby Union, 2020). The IRB developed a 10-year (2010–2020) strategic plan to develop rugby outside the eight founding nations, and the event was speculated to play a key role in its success (Wise, 2017). Japan prepared the event through involvement with various stakeholders, such as the Japanese government, relevant government offices (e.g., Japan Sport Agency and Japan Tourism Agency), local governments, Japan Sport Council, and private companies since 2009. According to the event report (The Japan Rugby Union, 2020), the number of tickets sold was 1.72 million, and the sold-out rate of the ticket was 99%, which was the highest ticket sales in the history of the Rugby World Cup. The number of views on social media related to the event reached 2.04 billion, which was more than five times higher than that of Rugby World Cup 2015 in England. Further, 13,000 volunteers were involved in the event, and the economic impact of the event generated a record-breaking JPY 646.4 billion (US $6.1 billion)^1^. Furthermore, the event itself generated a surplus of JPY 6.8 billion (US $65 million), being one of the most economically successful events in the history of the Rugby World Cup (EY, 2020). However, research on the social aspects and reports has been limited compared to the economic impact of the event.

### Data Collection

Tokyo residents participated in an Internet-based survey conducted by a Japanese Internet research service company. The panel data collection (i.e., the same sample in the pre- and post-events) occurred in August 2019 for the pre-event period (3 months before the event, T1) and March 2020 for the post-event period (4 months after the event, T2). For the timing of the data collection, a certain period was considered to avoid event-related euphoria (Gursoy and Kendall, 2006; Gibson et al., 2014), as well as the influence of the Tokyo 2020 Olympics and Paralympic Games, which had been scheduled for August 2020. Stratified sampling based on demographic variables (gender and age groups) from the Population Census of Tokyo was performed to establish a representative sample of 617 participants (successful response rate: 98.7%). Table 1 shows the descriptive statistics of the total sample and the participants. Among the respondents, 50.7% were male, the average age was 46.6 (SD = 15.3) years, 44.4% were employed full time, and 37.9% earned more than JPY 4,000,000 (Table 1). While their post-event interest in the Rugby World Cup 2019 was significantly higher than in the pre-event [*t*_(616)_ = 7.45, *p* < 0.001], no significant difference was found in the event support intention [*t*_(616)_ = 1.74, not statistically significant]. According to the Tokyo census, 49.2% of the population was male, and the average age was 45.02 years (Statistics of Tokyo, 2020). Small differences were found in gender (the male ratio was slightly higher) and average age (slightly higher) compared to the census data.

**Table 1:** Descriptive statistics of the total sample and the participants.

	**Total (*****N*** **=** **617)**		
Sex (% male)	50.7		
Age (mean and SD)	46.6 (15.3)		
Occupation (% employed full time)	44.4		
Marital status (% married)	54.5		
Personal income (% earning > 4,000,000 JPY/year)	37.9		
	**Time 1**	**Time 2**	***t*****-value**
Interest in RWC 2019	2.71 (1.54)	3.13 (1.75)	7.45^***^
How frequently do you think about RWC 2019	2.60 (1.59)	3.05 (1.83)	—
How interested are you in RWC 2019	3.01 (1.75)	3.45 (1.94)	—
How important is knowledge of RWC 2019 in your life	2.52 (1.55)	2.89 (1.75)	—
Event Support	3.41 (1.38)	3.33 (1.49)	1.74
I support RWC 2019 as a resident	3.10 (1.61)	3.12 (1.69)	—
Tokyo should bid for other major sporting events	3.72 (1.54)	3.53 (1.63)	—
Viewing behavior on TV (Did you watch RWC 2019 on TV?: Yes) (*n*/%)	—	305/49.4	—

*** *p < 0.001*.

### Measurements

*Social impact* was measured using a previously developed scale consisting of 20 items, representing six predetermined factors: “social cohesion” (SCOH: 4 items), “community spirit/feel good fact” (FGF: 3 items), “social capital” (SC: 4 items), “sport participation and physical activity” (SPA: 3 items), “disorder and conflict” (DC: 3 items), and “feelings of (un)safety” (FU: 3 items) (Taks and Rocha, 2017). The scale represents various previous social impact studies of sports events (e.g., Balduck et al., 2011; Gibson et al., 2014; Weed et al., 2015; Heere et al., 2016). For T1, expectations were framed in the future tense; for T2, items were frames in the past tense. Social impact items were measured on a seven-point Likert scale (from 1 = strongly disagree to 7 = strongly agree). In this scale, all items were worded in terms of “I” and/or “me,” representing the self-referenced social impact scale. Item scores were averaged to form an aggregate measure of each social impact construct.

The dependent variable *viewing behavior* was measured in T2 based on one item: “Did you watch Rugby World Cup 2019 on TV?” (dummy variable: yes = 1, no = 0). *Event support* was also measured in T1 and T2, using two items from previous studies (Ko and Stewart, 2002; Prayag et al., 2013), namely, “I support the Rugby World Cup 2019 as a resident” and “Tokyo should bid for other major sporting events.” These items were measured on a seven-point Likert scale (from 1 = strongly disagree to 7 = strongly agree) and averaged to form an aggregate measure of this construct. Internal reliability was acceptable with Cronbach α score of 0.70–0.76. Each of the two surveys was written in English and translated into Japanese, and the translation validity was verified by two native speakers.

### Analysis

Confirmatory factor analysis was performed by using AMOS 22.0 for both the expected and experienced social impact scales. Composite reliability (CR) and average variance extracted (AVE) values were computed for each construct to test convergent and discriminant validities. The comparative fit index (CFI ≧0.90), Tucker–Lewis index (TLI ≧0.90), adjusted goodness-of-fit index (AGFI ≧0.90), root mean square error of approximation (RMSEA ≦0.08), and standardized root mean square residual (SRMR ≦0.08) were utilized to confirm the goodness of fit (Hu and Bentler, 1999). We also assessed discriminant validity, which deals with a clear distinction between any pair of constructs, using the method suggested by Fornell and Larcker (1981). This method supports discriminant validity if the AVE value for each construct is greater than the squared correlation coefficients between the respective factors. To predict viewing behavior based on the pre-event expectations, a logistic regression analysis was performed. Multiple regression analysis was used to predict the event support intention by SPSS 22.0.

## Results

Table 2 shows that factor loadings for each item, as well as the CR and AVE for each factor pre- and post-event. The table also presents the results of the global fit indices that assess the proposed measurement model's fit with the data for both scales (pre- and post-event). The results of the global fit indexes, which assessed the proposed model's fit with the data in the pre- and post-event periods [pre-event: χ^2^/*df* = 2.75 (155), *p* < 0.000, CFI = 0.975, TLI = 0.970, AGFI = 0.911, RMSEA = 0.053, SRMR = 0.047; post-event: χ^2^/*df* = 3.01 (155), *p* < 0.000, CFI = 0.972, TLI = 0.966, AGFI = 0.901, RMSEA = 0.057, SRMR = 0.053] show that the measurement models fit the data well (Table 3). Moreover, the computed CR and AVE values for the 12 constructs (six expected and six experienced social impacts each) ranged from 0.71 to.95 for CR and from 0.45 to 82 for AVE; this indicates the reliability and convergent validity, but with some limitations (i.e., the recommended level for AVE is 0.50; Fornell and Larcker, 1981). Regarding discriminant validity, although most of the squared correlations did not exceed the AVE values, indicating discriminant validity in every construct, two pairs of factors (between expected/SCOH–experienced/SC in pre- and post-event) exceeded the AVE values (Table 3). Therefore, we compared the χ^2^ value of a measurement model with the correlation constrained to equal 1 to that of a baseline model without this constraint (Algesheimer et al., 2005). We performed a χ^2^ difference test for those pair of factors (for a total of two tests), and every case showed a significant difference (Δχ^2^ expected/SCOH–experienced/SC in pre = 81.03, Δ*df* = 1, *p* < 0.000; post = Δχ^2^ = 93.33, Δ*df* = 1, *p* < 0.000), suggesting that all measures of the constructs in the measurement model achieve discriminant validity. Therefore, the validity and reliability of this scale are acceptable.

Table 2Descriptive statistics of each scale (pre-event in future tense presented here; post-event—in past tense).

**Table:** 

**Constructs and Items**	**β**	**AVE**	**CR**
Social cohesion		0.76–0.74	0.93–0.92
1. The event will strengthen my friendships/relationships in the community	0.89–0.87		
2. The event will create my new friendships/relationships in the community	0.83–0.81		
3. The event will make me feel strongly connected to others	0.89–0.88		
4. The event will strengthen my sense of belonging in the community	0.87–0.88		
Community spirit/feel good factor		0.76–0.77	0.90–0.91
1. The event will increase my feelings of pride because Tokyo is hosting an event	0.80–0.83		
2. The event will increase my feelings of happiness because Tokyo is hosting the event	0.90–0.90		
3. The event will lift my spirits	0.91–0.90		
Social capital		0.76–0.82	0.93–0.95
1. The event will inspire me to become more engaged in the community	0.87–0.90		
2. The event will enhance my feelings of trust in the community	0.86–0.88		
3. The event will inspire me to more regularly attend community events	0.90–0.89		
4. The event will increase my social interactions in the community	0.85–0.94		
Sport participation and physical activity		0.77–0.79	0.91–0.92
1. The event will inspire me to become more involved in sport and/or physical activity	0.85–0.85		
2. The event will spark my interest in becoming more involved in sport and/or physical activity	0.89–0.92		
3. The event will increase my interest in sport and/or physical activity	0.90–0.89		
Disorder and conflict		0.45–0.53	0.71–0.77
1. The event will disturb my daily life in terms of peace and tranquility	0.73–0.78		
2. I will refrain from going to the city because it will be/is too crowded because of the event	0.66–0.74		
3. I will experience the event will cause traffic jams	0.61–0.65		
Feelings of (un)safety		0.69–0.69	0.87–0.87
1. I will feel unsafe because of potential terrorist attacks due to the event	0.86–0.89		
2. I will feel afraid that the event attracts terrorists	0.85–0.85		
3. I will be concerned about the increased levels of security due to the event	0.77–0.75

**Table:** 

	***χ***^**2**^ **/*****df***	**CFI**	**TLI**	**AGFI**	**RMSEA**	**SRMR**
Pre-event	2.75	0.975	0.970	0.911	0.053	0.047
Post-event	3.01	0.972	0.966	0.901	0.057	0.053

**Table 3:** Discriminant validity (average variance extracted value for each construct with the squared correlations between the respective constructs).

**(Pre-event)**	**SCOH**	**FGF**	**SC**	**SPA**	**DC**	**FUS**
SCOH	0.76					
FGF	0.69	0.76				
SC	0.83	0.69	0.76			
SPA	0.71	0.75	0.72	0.77		
DC	0.01	0.00	0.00	0.00	0.45	
FUS	0.00	0.00	0.00	0.00	0.40	0.69
**(Post-event)**	**SCOH**	**FGF**	**SC**	**SPA**	**DC**	**FUS**
SCOH	0.74					
FGF	0.53	0.77				
SC	0.86	0.55	0.82			
SPA	0.66	0.72	0.66	0.79		
DC	0.18	0.02	0.18	0.07	0.53	
FUS	0.07	0.00	0.07	0.03	0.41	0.69

Table 4 shows the results of the regression analysis for the effect of expected (pre-event) and experienced (post-event) social impact on, respectively, residents' viewing behavior and event support intention. The logistic regression results indicate that FGF and SPA have a significant positive association with viewing behavior, while SCOH has a significant negative association with viewing behavior. No significant role was found for other factors. The Hosmer–Lemeshow statistic for goodness-of-fit shows that the data fit the model well for the pre-event survey (χ^2^ = 13.32, *p* = 0.101). Additionally, the Nagelkerke *R*^2^ statistic shows that the predictor variables explain 36.9% of the variance for residents' viewing behavior. The multiple regression results in the post-event indicate that FGF, SC, and SPA have significant positive associations with the event support intention, whereas no significant role was found for the other factors. The adjusted *R*^2^ statistic shows that the predictor variables explained 77.2% of the variance for residents' event support intention. The variance inflation factor score of <10.0 indicates no multicollinearity concerns.

**Table 4:** Logistic regression and multiple regression results of the expected and experienced social impacts on the viewing behavior and event support intention.

	**Expected social impacts (viewing behavior)**		**Experienced social impacts (support intention)**	
	**β**	**Mean (SD)**	**β**	**Mean (SD)**
Social impacts				
1. Social cohesion	−0.451^*^	2.96 (1.37)	0.079	2.69 (1.37)
2. Community spirit/feel good factor	0.925^***^	3.51 (1.53)	0.390^**^	3.70 (1.67)
3. Social capital	−0.020	3.08 (1.40)	0.191^**^	2.79 (1.40)
4. Sport participation and physical activity	0.411^***^	3.36 (1.53)	0.298^***^	3.18 (1.59)
5. Disorder and conflict	0.152	3.94 (1.24)	−0.036	2.63 (1.27)
6. Feelings of (un)safety	−0.189	3.99 (1.34)	−0.011	2.98 (1.37)
*R^2^*	0.369^a^		0.772^b^	
Hosmer and Lemeshow	n.s.		—	

*Note*.

* *p < 0.05*,

** *p < 0.01*,

*** *p < 0.001*.

a *Nagelkerke R2*.

b *Adjusted R2*.

*n.s., not statistically significant*.

## Discussion

The logistic regression analysis showed that FGF, SPA (positively), and SCOH (negatively) were statistically significant predictors of viewing behavior. This implies that the expected social impact could be an effective predictor of consumer behavior, as indicated by previous studies (e.g., Hsu et al., 2010; Lee et al., 2011; Wong et al., 2013; Fan and Hsu, 2014). In particular, FGF, which corresponds to an emotional expectation (Gnoth, 1997), was a strong indicator of residents' viewing behavior. While consumer behavior could be explained by cognitive and emotional expectations (Gnoth, 1997; Perugini and Bagozzi, 2001), the results indicated that emotional expectation played a significant role in predicting viewing behavior for Rugby World Cup 2019. This effect might be caused by the emotional dominant aspects of sports products (Hall et al., 2010; Biscaia et al., 2012; Kim et al., 2017), as people could be motivated by the desire to have an emotional experience by viewing the event, which is consistent with the anticipated positive emotions playing an important role in their decision-making processes (Conner and Armitage, 1998; Perugini and Bagozzi, 2001; Yu, 2020). The results confirmed the expected emotional benefits of sports inspiring consumer behaviors.

SPA, which is a cognitive aspect of the social impact, was also a significant predictor of viewing behavior. This indicates that increasing interest in sports and physical activities through an event is a useful factor for residents' viewing behavior. This relationship might be explained by the importance of interest in sports to inspire sports consumer behaviors (Funk and James, 2001; Funk et al., 2002), which is a sport event–specific phenomenon. However, SCOH negatively predicted viewing behavior. Although the result is opposed to the effectiveness of social cohesion on event support (e.g., Balduck et al., 2011), the mean score was the lowest in the expected social impacts, which implies that it was the least expected benefit among all six. Furthermore, this might be caused by the characteristics of the dependent variable (i.e., viewing behavior), which is not necessarily accompanied by relationships with others, unlike on-site visiting behavior (i.e., they could watch the game alone). In any case, further investigation by replacing viewing behavior with visiting behavior, which requires more relationships with others, is necessary. Moreover, no significant influence of negative expected social impacts was found on viewing behavior [i.e., disorder and conflict and feelings of (un)safety]. This is possibly because individuals could watch the game on TV in their homes; these factors thus do not directly influence viewing behavior.

The multiple regression analysis showed that FGF, SC, and SPA positively predicted support intention. Similar to the results for Time 1 (pre-event), FGF and SPA played statistically significant roles in the event support intention. Furthermore, SC was a positive predictor for the intention, indicating that feelings of engagement, trust, and fostering interaction in the community due to the event are effective channels for residents to support the event. These results identifying the specific impacts on support intention could be more useful for obtaining event support than simply applying the summarized factors, such as perceived positive/negative impacts/benefits (e.g., Gursoy and Kendall, 2006; Lee and Krohn, 2013; Pappas, 2014; Gursoy et al., 2016; Al-Emadi et al., 2017; Ouyang et al., 2017; Parra-Camacho et al., 2020a), especially from the practical and/or managerial perspective. The event manager could thus focus on assets or marketing communication to obtain residents' support by emphasizing opportunities for FGF, SC, and SPA. Further, no significant effects of negative experienced social impacts were apparent. These mean values were low, even lower than before the event (Table 4), indicating that residents did not experience these factors as expected.

## Conclusions

This study mainly aimed to grasp the overall social impacts on host residents of Rugby World Cup 2019 by separating social impacts into expected (pre-event, T1) and experienced (post-event, T2) social impacts using the same panel data samples for both time intervals. The main contribution of this article is extending the understanding of the role of social impacts as predictor variables on residents' viewing behavior and event support intention through panel data to achieve more robust results (Menard, 1991). In particular, to the best of our knowledge, only few studies investigate the relationship between perceived social impacts and viewing behavior of sporting events. Considering the limitation of the explanatory power of consumer intention for actual behavior (Morwitz, 1997; Yoshida et al., 2013), clarifying the relationship between expected social impacts (pre-event) and actual viewing behavior during the event could provide valuable empirical evidence for the social impact literature. Specifically, the current self-referenced scale provides a more valid perspective of social impacts (Taks et al., 2020). Furthermore, this result extends the role of consumer expectation in the context of a major sporting event, the Rugby World Cup. Although the tourism literature has verified consumer expectancy and consumer intention and/or attitude (e.g., Hsu et al., 2010; Lee et al., 2011; Wong et al., 2013; Fan and Hsu, 2014), this study verifies the relationship between consumer expectancy and actual behavior, thus providing additional evidence of the expected role in actual consumer behavior.

Moreover, although many studies have used the summarized social impact (i.e., perceived impacts) to investigate its influence on support intention based on social exchange theory (e.g., Gursoy and Kendall, 2006; Lee and Krohn, 2013; Gursoy et al., 2016; Al-Emadi et al., 2017; Ouyang et al., 2017), current research identifies the significant role of FGF, SPA, and SC on residents' intentions. The results contribute to extending the social exchange theory in sporting events by identifying which impacts are more effective in forming residents' positive intention toward the event. Furthermore, this study reconfirms the importance of anticipated and/or experienced emotional impact on residents' behavior and intention as unique characteristics of sporting events (Hall et al., 2010; Biscaia et al., 2012; Kim et al., 2017). Therefore, event managers can inspire people's viewing behavior by stimulating the emotional aspects of the events and providing them with the opportunity to play sports and participate in physical activities. Indeed, the Rugby World Cup 2019 organization developed a campaign in 2019 to stimulate emotions through several communication channels (e.g., TV, Internet, SNS) and set up various stages in the streets among the host cities for people to play or experience rugby. This could be a useful marketing activity to obtain a higher viewing rate.

The main limitation of the current research is the lack of motivation factors in the current analysis. In addition to social impact expectations predicting consumer behavior, motivational factors play an important role (e.g., Hsu et al., 2010; Lee et al., 2011; Wong et al., 2013; Fan and Hsu, 2014). As motivation can be conceptualized into two variables, expectancy and intrinsic value (Heckhausen, 1989), further research must consider the introduction of motivation as another variable to explain residents' behavior. This seems a particularly useful pre-event, where the variance explained was lower than 0.40, leaving room for more explanatory factors to be included. Furthermore, our sample included only Tokyo residents. As the total number of host cities is 12, we could not conclude that the results represent all the host cities. Moreover, understanding will be further enhanced by separating the sample into, for example, low and high involvement groups or different segmentation groups (e.g., Ma et al., 2013; Parra-Camacho et al., 2020c). Finally, future research should develop a more comprehensive model describing consumers' full experience (pre–during–post) of the event by one model using panel data from the same sample pre- and post-event.

## Data Availability Statement

The raw data supporting the conclusions of this article will be made available by the authors, without undue reservation.

## Ethics Statement

The studies involving human participants were reviewed and approved by Tokai University. The patients/participants provided their written informed consent to participate in this study.

## Author Contributions

DO: conceptualization, methodology, investigation, data curation, writing-original draft, funding acquisition. SY: conceptualization, writing-review and editing. TF: conceptualization, methodology, and formal analysis. MT: writing-review and editing. All authors contributed to the article and approved the submitted version.

## Conflict of Interest

The authors declare that the research was conducted in the absence of any commercial or financial relationships that could be construed as a potential conflict of interest.

## Footnotes

^1^These results are reported based on standard economic impact analyses that do not consider the costs of hosting, as is the case for cost–benefit analyses; standard economic impact analyses are therefore often overestimations (Kesenne, 2012).
